# Natural sleep loss is associated with lower mPFC activity during negative distracter processing

**DOI:** 10.3758/s13415-020-00862-w

**Published:** 2021-01-20

**Authors:** Annika Dimitrov, Jonathan Nowak, Armin Ligdorf, Nicole Y. L. Oei, Mazda Adli, Henrik Walter, Ilya M. Veer

**Affiliations:** 1 Department of Psychiatry and Psychotherapy CCM, Charité – Universitätsmedizin Berlin, corporate member of Freie Universität Berlin, Berlin Institute of Health, Humboldt-Universität zu Berlin, Campus Charité Mitte, Charitéplatz 1, 10117 Berlin, Germany; 2 Department of Developmental Psychology, Addiction Development and Psychopathology-Laboratory, University of Amsterdam, Amsterdam, the Netherlands; 3 Amsterdam Brain and Cognition, University of Amsterdam, Amsterdam, the Netherlands

**Keywords:** Sleep loss, Working memory, Emotional distraction, Rostral anterior, Cingulate cortex, fMRI

## Abstract

Previous research has demonstrated that loss of sleep has a negative impact on both emotional and cognitive functioning. We examined whether subjectively reported natural sleep loss is associated with the interplay between emotion and cognition, as was probed by brain activity in response to emotional distraction during a working memory task. Forty-six healthy male adults reported their typical weekly sleep pattern using the Munich Chronotype Questionnaire (MCTQ), while recent sleep loss was enquired using a sleep diary in the 7 days preceding scanning. Participants performed a delayed match-to-sample task with negative and neutral distracters during the delay period inside the MRI scanner. Activity differences between negative and neutral distracters were associated to both sleep loss measures across participants. The amount of typically encountered sleep loss indicated by the MCTQ, but not sleep diary, was negatively associated with activity in the rostral anterior cingulate cortex and dorsomedial prefrontal cortex during emotionally negative compared to neutral distraction (*p* < 0.025, whole brain corrected). Participants showed less distracter-related activity in the ACC and dorsomedial PFC with increasing sleep loss, which, in the long run, might contribute to less adaptive emotional processing, and therefore a greater vulnerability to develop affective disorders.

## Introduction

Emotion and cognition depend on and influence each other, thus allowing adaptive and flexible human behavior (Joormann, 2019; Pessoa, 2008). This mutual dependence is apparent on a neuronal level as well, where “cold” cognition-related brain regions, such as the dorsolateral prefrontal cortex (dlPFC) and lateral parietal cortex (LPC), are functionally connected to “hot” affective regions, such as the amygdala and medial prefrontal cortex (mPFC) (Blair et al., 2007; Iordan, Dolcos, & Dolcos, 2013; Siciliano et al., 2017). A neurocognitive probe for the interaction between emotion and cognition are working memory tasks with emotionally negative or neutral distracters during the delay period. On the neural level, emotional distraction is reflected in stronger activity in emotion-related brain regions and decreased activity in regions related to executive functioning (Dolcos & McCarthy, 2006; Kalanthroff, Henik, Derakshan, & Usher, 2016; Schweizer et al., 2019; Shafer et al., 2012).

An important factor enabling fully functioning cognitive-emotional processing is adequate sleep. However, sleep deficit is a frequent finding in our around-the-clock society (Roenneberg, 2013), making individuals more vulnerable to impairments in emotional processing and executive functioning. This is supported by numerous neuroimaging studies (Gruber & Cassoff, 2014; Krause et al., 2017; Walker, 2009). In case of sleep deprivation, amygdala activation is typically elevated and its functional connectivity with prefrontal regions is impaired (Chuah et al., 2010; Motomura et al., 2013; Simon et al., 2015; Yoo, Gujar, Hu, Jolesz, & Walker, 2007). Although different studies in general report similar findings, the amount of sleep deprivation reported does differ considerably between studies (ranging from 35 hours of total sleep deprivation to 4 hours of sleep for 5 consecutive days). There also are notable inconsistencies in the patterns of brain activity changes due to sleep deprivation, which in part might stem from how emotional stimuli were presented, either as task-relevant or task-irrelevant (emotional distraction). Yoo et al. (2007) presented pictures with varying valence and reported enhanced bilateral amygdala activity to negative images, a decrease in functional connectivity between amygdala and mPFC, and an increase between the amygdala and brainstem regions after sleep deprivation. Finally Motomura et al. (2013) deployed an emotional face viewing task and reported an increase in left amygdala activity to fear images, whereas a decrease in functional connectivity was found between the amygdala and ventral ACC.

Chuah et al. (2010) administered a working memory task with faces as targets and emotional images as distracters. They reported enhanced bilateral amygdala activity to negative distracters and impaired functional connectivity to the dlPFC and ventromedial PFC (vmPFC). Simon et al. (2015) presented an N-Back task, in which the to-be-remembered numbers were superimposed on emotional distracter images. They found enhanced activity in the right dlPFC and left amygdala to neutral distracters after sleep deprivation, while functional connectivity between the amygdala and bilateral middle frontal gyrus was only found for well-rested participants. Besides deviating task paradigms, differences might in part stem from the choice of stimuli, as some studies included images of faces only, whereas other studies utilized complex scenes to elicit negative emotions.

In addition to the factors mentioned, there is a lack of studies examining loss of sleep under naturalistic circumstances, i.e., not artificially induced but as encountered in everyday life. Horne (2011) proposed that in a range of approximately 6-9 hours, sleep duration can be adapted to an individual’s needs, demands, and environment. Up to this point, however, it remains unclear how much sleep is necessary to maintain functioning in everyday life, considering that individuals have different sleep needs and patterns, which vary not only inter-individually (e.g., short and long sleepers) but also intra-individually (e.g., early work start or late night celebrations) (Goel, Basner, Rao, & Dinges, 2013).

Our purpose was to test how sleep deprivation under naturalistic circumstances affects brain regions involved in the interplay between cognition and emotion. In contrast to previous studies that applied artificial sleep deprivation, we assessed whether one’s typical sleep loss accumulated over the course of 1 week, either reported for a typical week or for the past 7 days before scanning, could be associated with differential brain activity during a working memory task with emotional distraction. We expected to find region-specific changes in brain activation during task performance due to emotional distracter presentation, specifically increases in emotion-related brain areas (e.g., amygdala, mPFC) and decreases in cognition-related areas (e.g., dlPFC), which should be more pronounced with accumulating sleep loss.

## Methods

### Participants

Participants were recruited via online and poster advertisement. We included male participants between the age of 18 and 48 years with relatively later sleep on- and offsets compared with the majority of the population, as was determined during the telephone screening interview (Roenneberg, Wirz-Justice, & Merrow, 2003). None of the participants reported any chronic physical diseases or a history of psychiatric disease (assessed by Structured Clinical Interview for DSM). The study was approved by the Medical Ethics Committee of Charité – Universitätsmedizin Berlin and complies with the ethical standards of the relevant national and institutional committees on human experimentation and with the Helsinki Declaration of 1975, as revised in 2008. Written informed consent was obtained from all participants. Fifty healthy male adults (*M* = 30.76 years; *SD* = 5.67; range 20-48) participated in this study, of which four had to be excluded from analysis due to structural brain abnormalities. Our final sample for analysis comprised 46 male participants (*M* = 30.15 years; *SD* = 5.42; range 20-48). Forty-two were right-handed, three left-handed, and one had no preference. However, all preferred to use the right hand for performing tasks at the computer and in the scanner. Scanning took place either on Wednesday (n = 25) or Thursday evenings (n = 21) between 5 pm and 10 pm. Participants refrained from caffeine 2 hours before scanning and from physical activity for the whole day.

### Assessment of sleep loss

Despite varying sleep needs and patterns, research on sleep loss always requires estimation of a baseline of sleep need, which bears a certain challenge in itself (Lauderdale, Knutson, Yan, Liu, & Rathouz, 2008; Miller et al., 2015). Some studies rely on subjective reports of sleep timings for several days, using a sleep diary, from which an average sleep duration can then be extracted. Other studies simply enquire one-time-only, how many hours of sleep one gets on average. In both cases, the indicated values rely on subjective perception, thereby potentially introducing bias. Whereas the sleep diary seems to be a more fine-grained and current assessment (state-like), it is uncertain how representative, and therefore generalizable, this sleep pattern is for other periods of time. On the other hand, an average estimation of sleep duration should encompass weekly variations (trait-like), although the reliability of the estimation of one’s sleeping behavior and its relatedness to other study variables might be questioned.

For assessment of trait-like sleep loss, the Munich Chronotype Questionnaire (MCTQ; Roenneberg et al., 2003) was used within the context of the telephone screening. The MCTQ assesses sleep duration for typical work (SDw) and free days (SDf) separately, by enquiring participants’ mean sleep on- and offsets. From these parameters, the midpoint of sleep can be determined (Table 1). In this study, we included participants who had a midpoint of sleep later than 4:30 a.m., which could be considered later intermediate chronotypes and late chronotypes (Roenneberg et al., 2007). We focused on this sample in particular, as people with an intermediate to late chronotype more often show sleep loss (Roenneberg & Merrow, 2007). Following Roenneberg et al. (2003), the following formula was used to assess sleep loss if SDw was smaller than the mean sleep duration for the whole week (SDm): (SDm-SDw)*(work days per week) (n = 43). However, if SDw was larger than SDm (n = 3), the following formula was applied: (SDm-SDf)*(free days per week). The resulting number indicates an absolute value of how many hours of sleep are lost, either on work or free days for a typical week. In contrast, for assessment of state-like sleep loss, we asked participants to keep a sleep diary 7 days before scanning, additionally indicating which of the 7 days were work or free days. In analogy to the MCTQ, we extracted SDm, SDw, SDf, and sleep loss from the diary, averaged across the specified number of work and free days. However, two participants had to be excluded from state-like sleep loss analysis, because no free days were indicated in the sleep diary, although during the telephone screening participants reported a 5-day working week. By using both the MCTQ and sleep diary data, we expected a deeper understanding for the possible consequences of choosing a trait-like or state-like sleep loss measurement.

**Table 1:** Sleeping parameters that were assessed using the Munich ChronoType Questionnaire (MCTQ)

Variable	Format	Abbreviation	Computation
No. of work days/free days per week	n	WD, FD	-
Sleep onset/end on work days/free days	hh:mm	SOw, SOf / SEw, SEf	-
Sleep duration on work days/free days	hh:mm	SDw / SDf	SDw = SEw – SOw SDf = SEf - SOf
Average weekly sleep duration	hh:mm	SDm	(SDw x WD + SDf x FD)/7
Weekly sleep loss from MCTQ/sleep diary	hh:mm	SLoss (MCTQ) / SLoss (diary)	If SDm > SDw: (SDm - SDw) x WD If SDm ≤ SDw: (SDm - SDf) x FD
Mid-sleep on free days	hh:mm	MSF	SOf + SDf/2
Chronotype (MSF sleep corrected)	hh:mm	MSF_sc_	If SDf ≤ SDw: MSF If SDf > SDw: MSF - (SDf - SDm)/2

### fMRI task

**Fig. 1:**
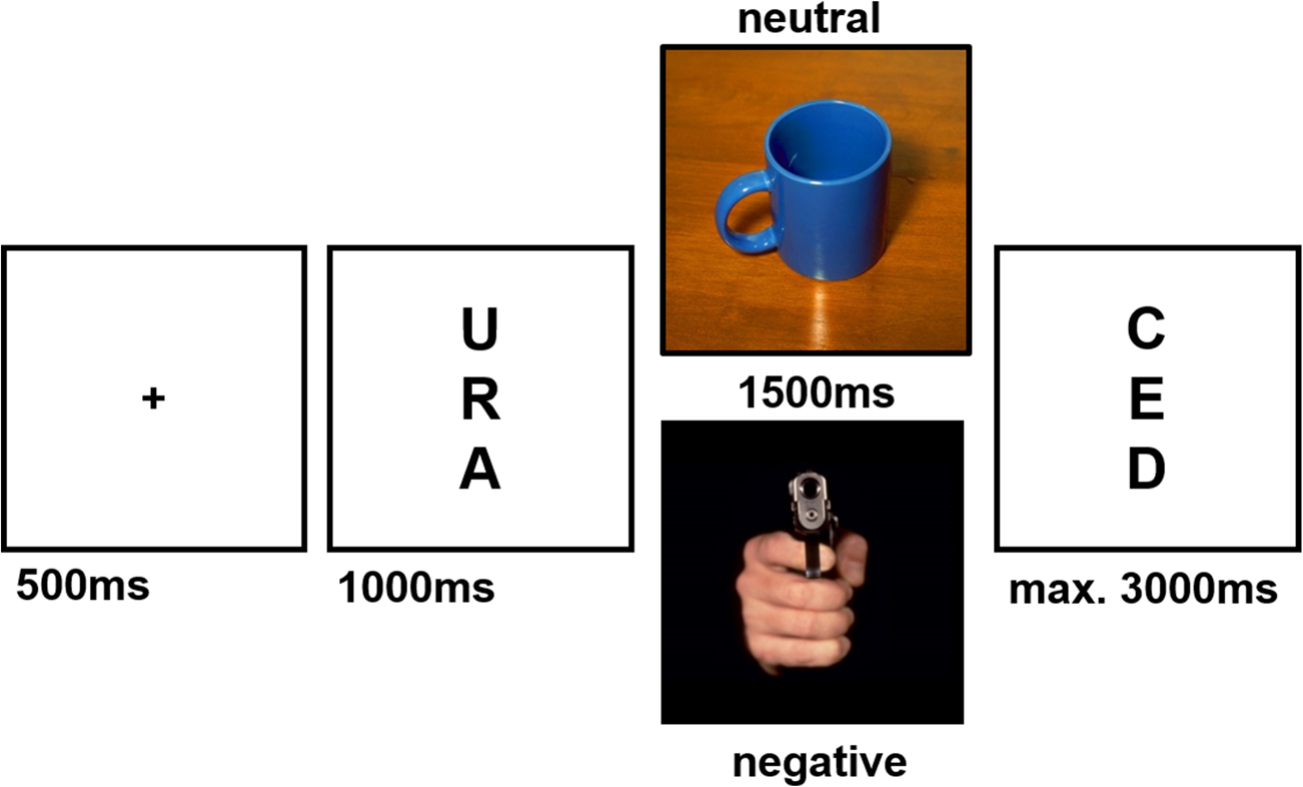
Example trial of the Sternberg task with distracters during delay interval (upper distracter: neutral, lower distracter: negative). ms, milliseconds

Participants were scanned while performing a Sternberg working memory task with negative and neutral distracters (International Affective Picture System; Lang, Bradley, & Cuthbert, 2008) during the delay period (Oei, Tollenaar, Spinhoven, & Elzinga, 2009). The task consisted of 48 trials (24 neutral, 24 negative) in an event-related design, each starting with a fixation cross (500 ms), followed by a target display of three letters (1,000 ms), a neutral (norm ratings: valence: *M* = 5.01 ± 1.19, arousal: *M* = 2.71 ± 1.93) or negative (norm ratings: valence: *M* = 2.09 ± 1.45, arousal: *M* = 6.48 ± 2.22) distracter (Lang et al., 2008) during the delay interval (1,500 ms), and finally a probe display of three letters (maximum 3,000 ms), in which any of the target letters could be present or absent (24 trials each; see Fig. 1). The inter-trial interval was jittered between 1,500 and 4,500 ms. If one of the target letters was present in the probe letters, a button had to be pressed with the index finger. If the target letter was absent, a second button had to be pressed with the middle finger. Reaction times and accuracy were recorded. Directly before scanning, participants were presented ten practice trials with neutral images to become familiar with the task. Because we only had one experimental and no control group, the order of trials was the same for each participant so that possible order effects applied equally to each participant, whereas the order of trials itself was pseudorandomized. The software suite Presentation (Neurobehavioral Systems, Inc.) was used for task presentation.

The emotional distraction task was embedded in a longer scanning protocol. It was preceded by a structural scan, a first resting state scan, a social stress task, and a second resting state scan and was followed by spectroscopy. Results from these scans will be reported elsewhere. The total scan duration was 90 minutes. In order to assess physiological stress responses, saliva samples as well as subjective stress ratings on a scale from 1 to 10 were taken at several timepoints during the protocol. For this study, we focused on the period before (t1 = pre-rest 1, t2 = pre-stress) and directly after the stressor (t3 = post-stress, t4 = post-rest 2, t5 = post emotional working memory task), which should best indicate a stress response. After scanning, participants completed the following questionnaires to ensure psychological wellbeing: Beck Depression Inventory II (Beck, Steer, and Brown (2009)), Perceived Stress Scale (Cohen, Kamarck, and Mermelstein (1983)), State-Trait-Anxiety-Inventory trait version (Spielberger, Gorsuch, Lushene, Vagg, and Jacobs (1983)), Positive and Negative Affect Scale (Watson, Clark, and Tellegen (1988)), and Childhood Trauma Questionnaire (Wingenfeld et al. (2010)).

### Behavioral analysis

Behavioral output from the emotional distracter task was checked for the amount of errors and outliers. If reaction times exceeded 3 standard deviations from the mean, they were classified as outliers and values were replaced by *M* ± (3*SD). Next, for accuracy and reaction times a 2 (target type: present or absent) x 2 (distracter type: negative or neutral) repeated measures ANOVA was done with sleep loss, either as assessed by MCTQ or sleep diary, as covariate of interest, while controlling for age. Greenhouse-Geisser corrections were applied when the assumption of sphericity was not met. All behavioral analyses were performed with IBM SPSS Statistics for Windows, Version 22 (IBM Corp., Armonk, NY).

### fMRI data acquisition

Scanning took place on a 3T Siemens Trio scanner with a 32-channel head coil. For the emotional distraction task, we acquired GE-EPI images with the following parameters: TR = 1,560 ms, TE = 25 ms, and flip angle = 65°. Twenty-eight slices of 3-mm isotropic voxels were acquired sequentially in descending order, auto-aligned parallel to AC-PC line. The slices stack was positioned so that all of the temporal lobe was included, which resulted in exclusion of dorsal parts of the brain in some participants due to the restricted number of slices that could be acquired given our TR. Acquisition time of the total task, and hence the number of volumes, which depended on how fast participants responded to the probe, ranged from 5:26 min to 6:35 min (i.e., 209 to 253 volumes). For registration purposes, we acquired a high-resolution structural T1 (MPRAGE; 1-mm isotropic voxels, TR = 1,900 ms, TE = 2.52 ms, flip angle = 9°) and a Fieldmap image (3-mm isotropic, TR = 434 ms, TE = 5.19 ms, flip angle = 60°).

### fMRI data preprocessing and analysis

The following preprocessing was carried out using FSL (Smith et al., 2004): motion correction, slice-time correction, brain extraction, and spatial smoothing with a FWHM of 6mm. Registration parameters were obtained for the functional-to-structural transformation, using the Fieldmap to correct for inhomogeneity artifacts (FSL’s epi_reg command, employing the Boundary Based Registration [BBR] algorithm). Normalization parameters for the structural-to-standard-space (2mm MNI) transformation were obtained with Advanced Normalization Tools (ANTs; Avants et al., 2011). Next, functional data were further cleaned from artifacts using ICA-AROMA (Pruim et al., 2015), which regresses out latent signal sources (independent components) that it classifies as noise. Lastly, a high-pass temporal filter of 125 s was applied to the cleaned 4D images, which were then normalized to standard space using the previously derived registration parameters.

First-level analyses were performed with FSL’s FMRI Expert Analysis Tool (FEAT v6.00). A general linear model was set up and tested for each participant, including four regressors: 1) negative (neg) distracter; 2) neutral (neu) distracter; 3) probes; and 4) error trials. All were convolved with a double-gamma HRF, and temporally filtered with a high-pass filter of 125 s, similar to the functional data. Distracter events were modeled to span the period from the onset of the target to the end of the distracter. Probe events spanned the onset of the probe to the response. Error trials spanned the length of the entire trial, from onset of the target to the response. The contrast of interest was neg vs. neu distracters. Activity differences between negative and neutral distracters were assessed with a one-sample *t*-test, as was the association of these differences with sleep loss, including age as covariate. The resulting *t*-statistical maps then underwent Threshold-Free Cluster Enhancement (TFCE; Smith & Nichols, 2009), using the default parameter settings (H = 2, E = 0.5, C = 6), and significance testing was performed with permutation testing (4,000 iterations) using the in-house developed TFCE_mediation software (Lett et al., 2017). In the latter step, a null distribution of random results was generated against which the empirical findings were tested, which resulted in statistical images that are family-wise error corrected for multiple comparisons at *p* < 0.05 for the main effect of distracter type, and at *p* < 0.025 for the two sleep loss variables (i.e., MCTQ and sleep diary). Furthermore, a Region-of-Interest (ROI) mask was created, based on previous literature concerning sleep loss and emotional distraction tasks, using the Harvard-Oxford (Sub-)Cortical Structural Probability Atlas. Included were the anterior cingulate gyrus, the frontal medial cortex, the middle frontal gyrus, and bilateral amygdala, each thresholded at the lowest probability to include all voxels that could be associated to these regions. The ROI mask, as well as the voxelwise uncorrected (*t*) and corrected (TFCE *p*) statistical maps of our analyses are available on NeuroVault.org (Gorgolewski et al., 2015) via this link: http://neurovault.org/collections/3428.

## Results

The typical amount of sleep loss reported by our participants on the MCTQ varied between 0 and 7:30 hours per week (*M* = 2:26; *SD* = 2:05; n = 46). In comparison, the total sleep loss reported during the week before scanning varied between 0:13 and 9:48 hours (*M* = 1:55; *SD* = 1:49; n = 44). Intraindividual variability in sleep duration within a subject across seven nights varied between 0:23 and 3:23 hours (*M* = 1:05; *SD* = 0:33; n = 46). Whereas sleep loss from the MCTQ did not show any outliers in the explorative analysis, sleep loss from the sleep diary included two moderate (≥1.5 standard deviation) and one extreme outlier (≥3 standard deviation). For an overview see Table 2.

**Table 2:** Descriptive statistics for sleep variables

	MCTQ				Sleep diary							
	SDw	SDf	SDm	SLoss	SDw	SDf	WD	FD	SDm	SLoss	SD7	SDvar
	hh:mm				hh:mm		n		hh:mm			
n	46	46	46	46	46	44*	46	46	46	44*	46	46
*M*	6:43	8:21	7:11	2:26	6:57	7:49	5	2	7:13	1:55	6:58	1:05
*SD*	1:01	1:10	0:48	2:05	1:04	1:23	0.63	0.63	0:55	1:49	1:16	0:33
Min	4:25	5:40	4:46	0:00	3:38	4:15	3	0	4:40	0:13	2:36	0:23
Max	8:30	11:00	8:23	7:30	9:12	11:45	7	4	9:22	9:48	10:30	3:23

*n = 2 missing as indicated no free days in week before scanning, *M* = mean value, *SD* = standard deviation, Min = minimum value, Max = maximum value, SDw = sleep duration during work days, SDf = sleep duration during free days, SDm = average sleep duration across 7 nights, SLoss = sleep loss, WD = work days a week, FD = free days a week; SD7 = sleep duration during night before scanning; SDvar = variability in sleep duration across 7 nights; hh:mm = hours:minutes.

Both sleep loss variables showed no association with age (both *p* > 0.05). Sleep loss as measured by MCTQ and by sleep diary were positively associated (*r*_*S*_ = 0.44, *p* = 0.003). A Mann-Whitney *U* test indicated no significant difference in sleep loss depending on which day was scanned (both *p* > 0.05). No reaction time outliers were detected.

Mean value of the BDI was 4.09 points (±3.83; range 0-15 points), confirming that our participants did not show clinically significant signs of depressive symptoms. There was no association between the two sleep loss measures and BDI score (*p* > 0.05), also not when excluding item P, which enquires sleep problems.

### Behavioral findings

#### Accuracy

**Fig. 2:**
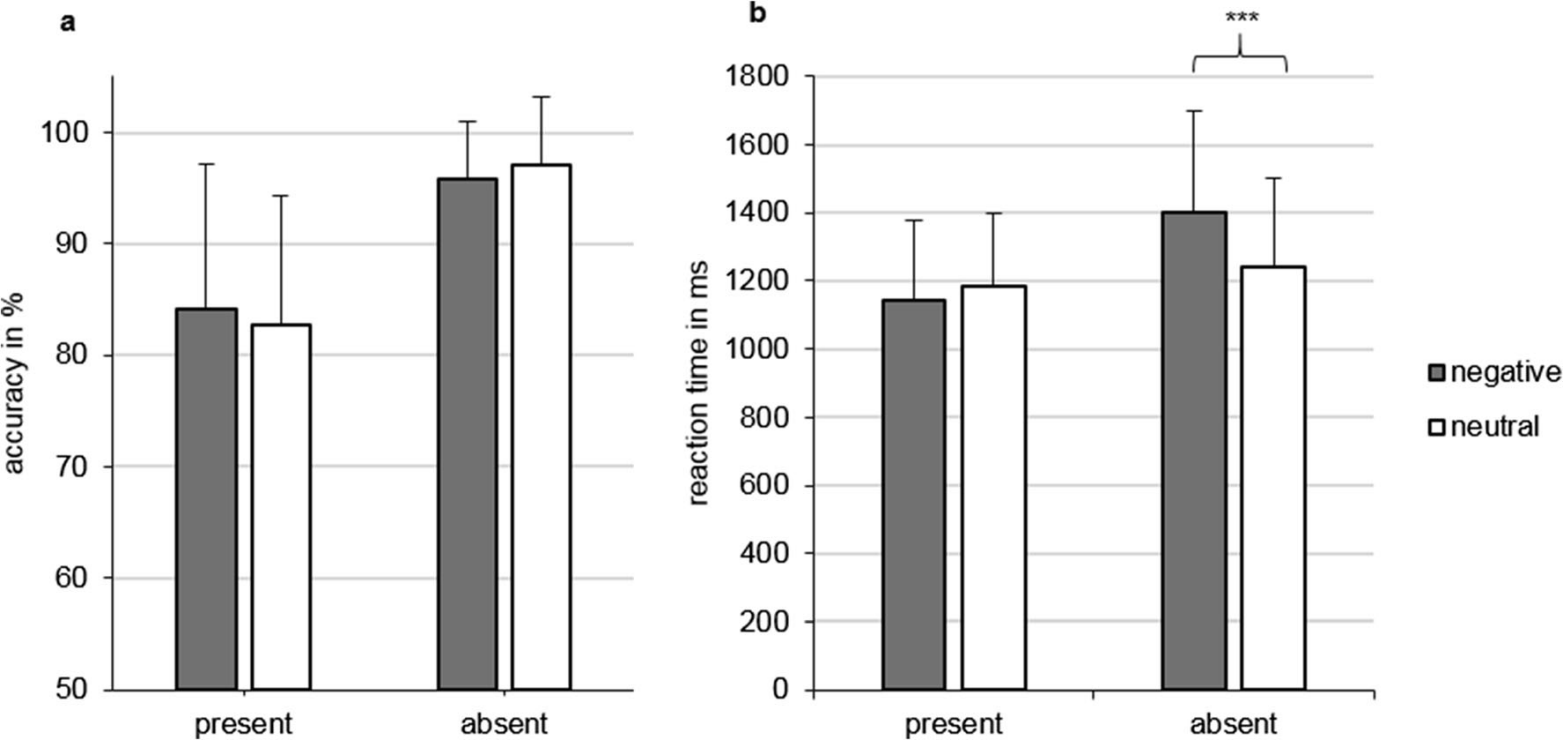
**a** Accuracy rate (in percent) for the negative and neutral distracter conditions and for present and absent responses. **b** Mean reaction times (in ms) for the negative and neutral distracter conditions and for present and absent responses. ***p < 0.001; ms, milliseconds

Mean accuracy (Fig. 2a) did not differ between negative (90.04% ± 7.05) and neutral distracters (89.95% ± 6.67), or between present (83.51% ± 9.98) and absent (96.47% ± 3.62) trials (all *p* > 0.05). A significant interaction was observed between probe and MCTQ sleep loss (*F*(1, 43) = 4.141, *p* = 0.048, partial *η*^*2*^ = 0.088): Higher accuracy was associated with less sleep loss on present trials, while this was not found for absent trials. There was no association between accuracy and sleep diary sleep loss (*p* > 0.05). Age showed a significant between-subjects effect for MCTQ sleep loss (*F*(1,43) = 5.364, *p* = 0.025), whereas age showed a trend for sleep diary sleep loss (*F*(1,41) = 4.017, *p* = 0.052).

#### Reaction times

Mean reaction times (Fig. 2b) did not differ between negative (1274.14 ms ± 250.69) and neutral (1213.46 ms ± 218.69) distracters, or between present (1165.34 ms ± 207.83) and absent (1322.25 ms ± 272.03) trials (all *p* > 0.05). There was a significant interaction (Fig. 1b) between valence and probe (MCTQ: *F*(1,43) = 7.289, *p* = 0.01, partial *η*^*2*^ = 0.145 ; sleep diary: *F*(1,41) = 5.839, *p* = 0.02, partial *η*^*2*^ = 0.125), indicating that in present trials there was no difference between neutral (MCTQ: 95% confidence interval [CI] 1122.29–1248.42, sleep diary: 95% CI 1115.77–1249.27) and negative distracters (MCTQ: 95% CI 1077.98–1212.68, sleep diary: 95% CI 1063.21–1203.88), whereas in absent trials participants responded faster to neutral (MCTQ: 95% CI 1163.94–1319.18, sleep diary: 95% CI 1161.11–1321.93) compared with negative distracters (MCTQ: 95% CI 1313.99–1491.90, sleep diary: 95% CI 1308.28–1492.95, *p* < 0.001). No interaction was found between reaction times and sleep loss (both MCTQ and sleep diary: *p* > 0.05). No effect was found for the covariate age (*p* > 0.05).

### fMRI findings

#### Main effect of distracter type

**Fig. 3:**
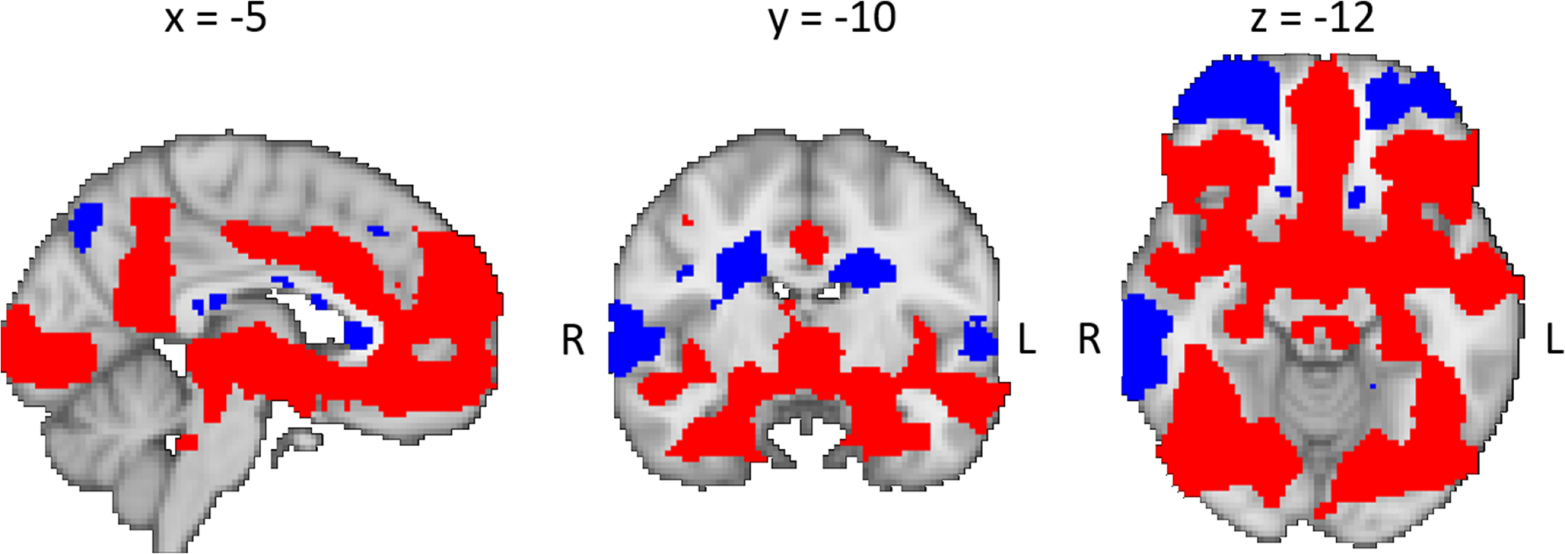
Main effect across group for the contrast negative > neutral distracter (*p* < 0.05, TFCE-corrected for multiple corrections) overlaid on the 2-mm MNI template. Red indicates negative > neutral, while blue indicates neutral > negative distracter. R, right; L, left

Contrasting negative and neutral distracters across all participants demonstrated activity differences in a distributed set of brain areas, as can be appreciated in Fig. 3 (*p* < 0.05, TFCE-corrected for multiple comparisons). Regions that were more strongly activated during negative than neutral distracters included the amygdala, hippocampus, rostral and dorsal anterior cingulate gyrus, ventromedial PFC, dorsomedial PFC, middle temporal gyrus, and occipital cortex, whereas more activity was found for neutral than negative distracters in the ventrolateral PFC, dorsolateral PFC, angular gyrus, and superior and inferior temporal gyri.

#### Associations with sleep loss

**Fig. 4:**
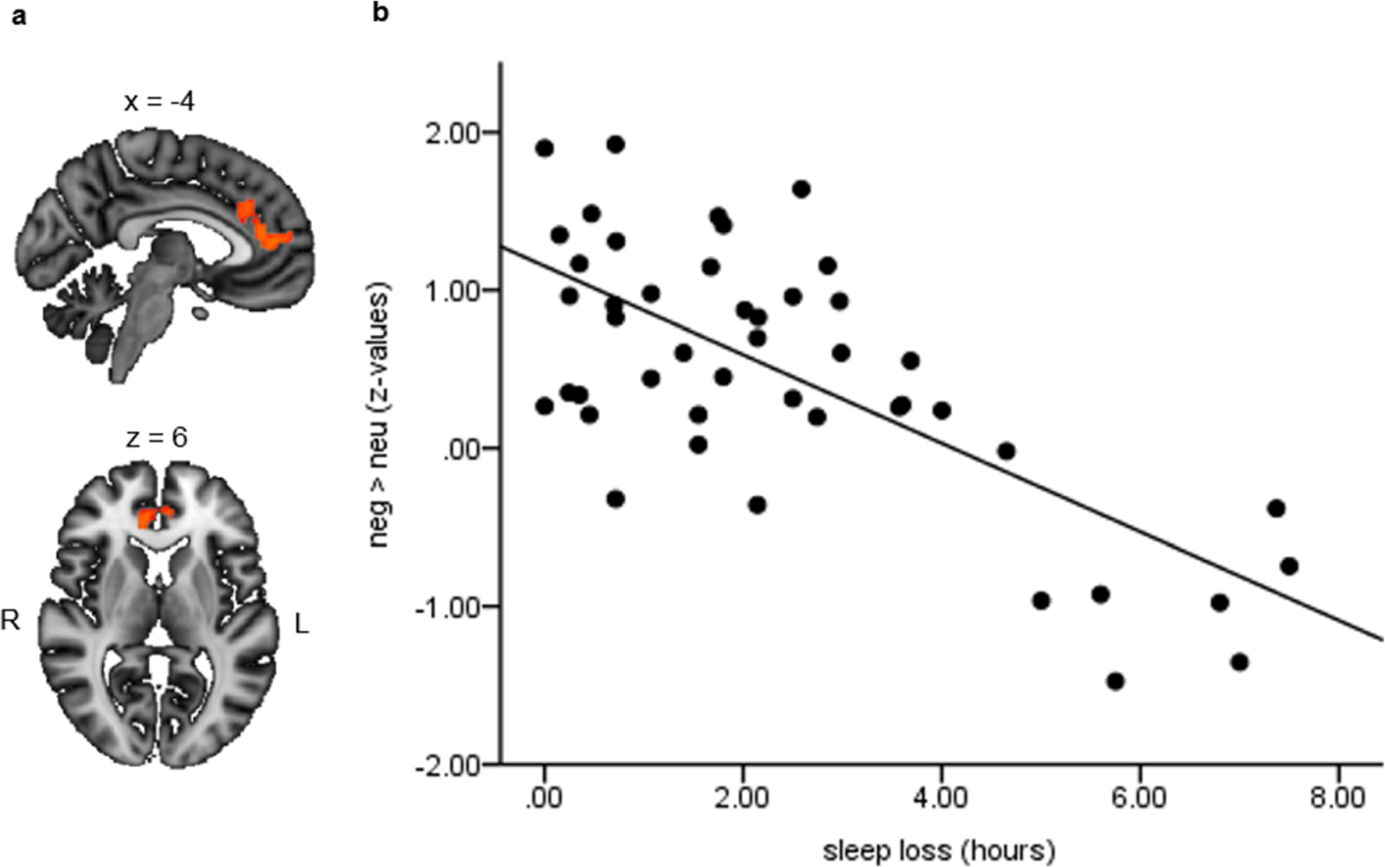
**a** Activity in the anterior cingulate cortex and dorsomedial prefrontal cortex for the contrast negative > neutral is associated with the amount of sleep loss estimated by the MCTQ (*p* < 0.025, TFCE-corrected for multiple corrections). **b** Scatter plot illustrating the association between negative > neutral distracter activation and sleep loss in the rostral ACC cluster. R, right; L, left; neg, negative; neu, neutral

In case of the whole-brain analysis, activity in the rostral anterior cingulate cortex (rACC; peak MNI coordinates: x = −2, y = 46, z = 10, cluster size = 522 voxels) and parts of the dorsomedial PFC (dm PFC; peak MNI coordinates: x = 0, y = 64, z = 28, cluster size = 19 voxels) were negatively associated with the amount of MCTQ sleep loss (*p* < 0.025, TFCE-corrected for multiple comparisons). That is, participants with less sleep loss tended to have higher activation in these regions during emotional than neutral distracters, whereas the opposite pattern was found for participants reporting higher levels of sleep loss (Fig. 4). No significant association was found for sleep loss in the week preceding the scan session, as reported in the sleep diary kept by the participants. However, we found a similar negative association within the rACC at a lenient uncorrected threshold (*p* < 0.001). As for the ROI analysis, significant associations overlapped with the results of the whole-brain analysis.

#### Exploratory post-hoc analysis

Furthermore, instead of the average sleep loss over the seven days prior to scanning, it may rather be intraindividual variability in sleep duration across these days that is associated to the task activity. Here, variability was determined by calculating the standard deviation of the sleep durations reported for each of the seven days spanning the sleep diary. However, we did not find a significant association between sleep variability and task activity (*r* = −0.15; *p* = 0.31). Yet, sleep duration during the night before scanning might prove more sensitive to predict mPFC activity in response to emotional distracters than the variability of the average sleep duration over the week. We indeed found such an association (*r* = 0.31; *p* = 0.037).

Three participants stated to sleep longer on work than on free days, which lead to the calculation of sleep loss based on another formula compared with the other 43 participants. To ensure that those values did not influence our results, we correlated task activity in the rACC with sleep loss for the complete sample and for the 43 participants with sleep loss on work days only. Results showed a similar Spearman’s *r* (n = 43: *r* = −0.549, *p* < 0.001; n = 46: *r* = −0.549, *p* < 0.001).

Last, to rule out that sleep loss magnified stress effects, we conducted a repeated measures ANOVA on the subjective stress ratings and cortisol concentrations over the course of the experiment, with sleep loss as predictor and age as covariate. There was no effect for either recent (sleep diary) or trait-like sleep loss (MCTQ) on the subjective stress ratings or cortisol concentrations. On the other hand, to examine whether stress induction might have influenced sleep loss effects, we correlated the cortisol area under the curve with respect to increase (AUCi; Pruessner, Kirschbaum, Meinlschmid, & Hellhammer, 2003) with task activity in the rACC, which resulted in a nonsignificant association (*r*_*s*_ = 0.055, *p* = 0.727, n = 43, t_1_ – t_4_). Furthermore, we conducted two simple mediation analyses with PROCESS for SPSS V3.5 (Hayes, 2017) to explore whether AUCi or cortisol concentrations directly before the emotional working memory task mediated the association of sleep loss with mean activity changes in the rACC. First, we incorporated AUCi as a mediator and age as a covariate. A significant effect of sleep loss on rACC activity was observed (B = −0.29, *p* < 0.001, n = 43). We did not observe an indirect effect (*p* > 0.05). Thus, AUCi did not mediate the association between sleep loss and rACC activity. Likewise, when taking into account the cortisol concentrations at t_4,_ the association of sleep loss with rACC activity resulted in a significant direct effect (B = −0.29, *p* < 0.001, n = 45), whereas cortisol concentrations on t_4_ did not mediate this association (*p* > 0.05).

Finally, we tested whether the association of sleep loss with rACC activity depends on being a cortisol responder. We defined the status of being a cortisol responder by a 15% increase of cortisol concentrations from directly before stress (t_2_) in relation to t_3_ or t_4_, based on which time point resulted in a higher cortisol concentration (Miller, Plessow, Kirschbaum, & Stalder, 2013). Twenty-three participants were classified as cortisol responders and 21 participants as nonresponders. We performed a Mann-Whitney *U* test to evaluate possible differences in sleep loss and brain activity, which were both nonsignificant (*p* > 0.05).

**Fig. 5:**
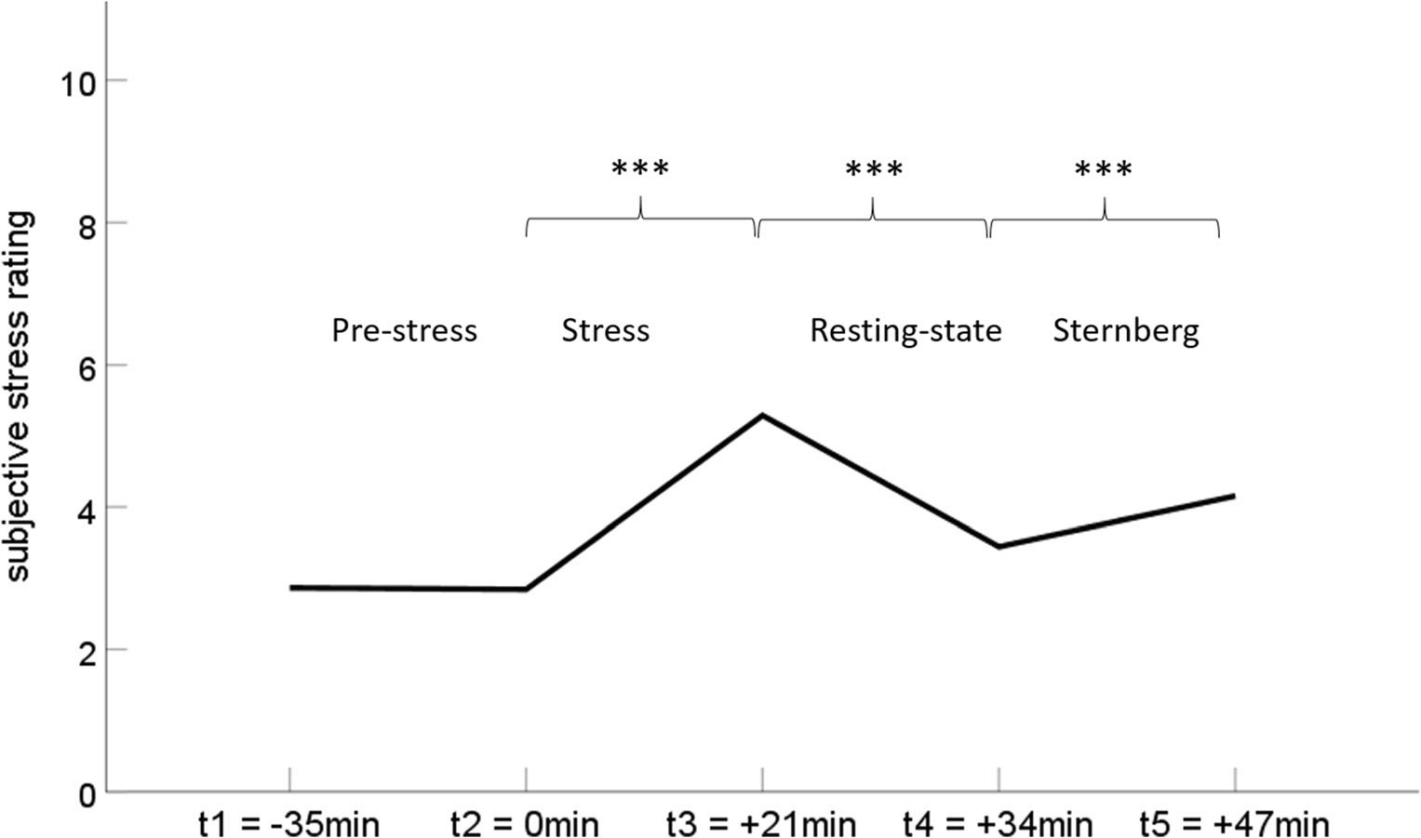
Timeline of subjective stress ratings (***p ≤ 0.001)

Interestingly, the progression of the subjective stress ratings displayed a significant increase in Wilcoxon signed-rank test from before to directly after stress (t_2_ to t_3_, *z* = 5.537, *p* < 0.001), a significant decrease from directly after stress to after resting state (t_3_ to t_4_, *z* = 3.302, *p* = 0.001) and again a significant increase during the emotional distracter task (t_4_ to t_5_, *z* = −5.327, *p* < 0.001) (Fig. 5).

## Discussion

In the present study, we examined the associations of naturally occurring sleep loss in healthy men with brain activity changes during a delayed match-to-sample working memory task with emotional distracters presented during the delay period. Our results support previous findings concerning emotional distraction; Stronger activity was found in “hot” affective brain regions when presented with negative compared with neutral images (Dolcos & McCarthy, 2006; Iordan et al., 2013; Oei et al., 2012). These regions included, amongst others, bilateral amygdala, hippocampus, medial PFC, cingulate cortex, and occipital cortex. In contrast, relative deactivations were observed in “cold” cognition-related brain regions, namely the dorsolateral PFC and angular gyrus, which are regions known to be involved in executive processing. For sleep loss accumulated over the course of an average work week, as was estimated by the MCTQ, our data revealed an association with activity changes in the rostral anterior cingulate cortex (rACC) and parts of the dorsomedial PFC: the higher the amount of sleep loss reported, the less activated the aforementioned regions were during emotional distraction. Both regions are known to be involved in affective processes and play an important role in regulating limbic regions (Etkin, Egner, & Kalisch, 2011; Margulies et al., 2007).

Several studies provide support for involvement of the rACC in syndromes that feature symptoms in the domains of sleep and emotion processing. For example, one study reported an increase in rACC volume in patients with chronic primary insomnia, a disorder that is known to be comorbid with depression (Winkelman et al., 2013). In a meta-analysis, hypoactivation in the rACC appeared as the predominant neural activation pattern during cognitive-emotional challenge tasks in patients with major depression (Diener et al., 2012). However, not only depression is associated with altered rACC activity. A study on bipolar disorder in adolescents found a positive association between average sleep duration and rACC activity in healthy controls during a cognitive control task, but a quadratic relationship for bipolar patients (Soehner, Goldstein, Gratzmiller, Phillips, & Franzen, 2018), indicating that both short and long sleep were associated with greater rACC activity in patients.

Likewise, the medial PFC has been shown to play a pivotal role in emotion- and self-regulative processes. Dörfel et al. (2014) compared neural activations of four different emotion regulation strategies (detachment, reinterpretation, distraction, expressive suppression) and found a common set of brain regions, including the dorsomedial PFC, bilateral dorsolateral PFC, inferior parietal cortex, and anterior insula. In a review by Ochsner and Gross (2005) about the cognitive control of emotion, they report that the ACC and medial PFC are especially important for attentional distraction during presentation of emotional stimuli. Furthermore, several studies observed a decrease in functional connectivity between amygdala and medial PFC due to sleep deprivation (Chuah et al., 2010; Motomura, Katsunuma, Yoshimura, & Mishima, 2017; Yoo et al., 2007), supporting the assumption that sleep loss might lead to decreased cognitive control in regulating emotional processing. On a structural level, a clinical study reported that lower sleep quality was associated with a decrease in medial PFC volume in patients with chronic fatigue syndrome (Shan et al., 2017).

Previous findings suggest a strong relationship between sleep and emotional processing, which may already emerge through a naturally induced state of sleep restriction. Furthermore, the mPFC plays an important role in emotional processing and affective disorders, but at the same time, it is related to sleeping patterns and disorders, thereby suggesting that mPFC areas function as important hubs between the two domains. One should keep in mind that most people will compensate for lost sleep on free days, thereby returning to a homeostatic state (Borbély, Daan, Wirz-Justice, & Deboer, 2016) and remaining resilient against the development of physical or psychological disorders (Kalisch et al., 2017). Future research therefore should focus on potential protective mechanisms that could prevent individuals from suffering from sleep loss.

We did not find an impact of sleep loss on performance in terms of reaction times (RT) and accuracy, when comparing emotional and negative distracter images. Because previous studies did not report performance results in a consistent way, we cannot readily compare our findings to those published before. Yoo et al. (2007) and Motomura et al. (2013), for example, conducted passive tasks without any accuracy or RT monitoring. Both Simon et al. (2015) and Chuah et al. (2010) found lower accuracy for neutral and emotional distracters after sleep deprivation but did not report on the impact of sleep loss on RTs. In the current study, we did not find differences in performance due to sleep loss. An explanation could be offered by the relatively moderate processing load of our working memory task (i.e., three letters for both target and probe), which could have caused a ceiling effect in our sample. Therefore, inclusion of trials with higher processing loads might be better suited to reveal behavioral differences (Oei, Everaerd, Elzinga, van Well, & Bermond, 2006).

Because the amount of sleep loss in our study was significantly lower compared with other studies, it might be that behavioral consequences only become apparent after profound sleep loss, as more subtle sleep loss may be compensated for by other mechanisms, such as increased vigilance. Youngstedt et al. (2009) provided support for this notion, demonstrating that sleep restrictions of 90 minutes per night for 10 consecutive weeks did not lead to changes in neurobehavioral performance in older adults. Rupp, Wesensten, and Balkin (2012), as well as Mu et al. (2005), even posited that vulnerability to sleep loss is a trait-like characteristic, hence discriminating between individuals who will perform similarly independent of the amount of sleep and individuals who are more sensitive to the effects of sleep loss. On the other hand, Horne (2011) argued that humans can adapt to shorter or longer sleep durations within a range of approximately 6-9 hours without increased daytime sleepiness or negative health consequences. Although we generally describe our participants as sleep-deprived, we would like to point out that 14 participants reported less than 1 hour of sleep loss, while two of them reported no sleep loss at all, which translates to not being sleep deprived. However, we decided not to exclude those participants in order to represent the natural variability of possible sleep loss values on a continuum.

When we repeated analysis of neuroimaging data with the more state-like sleep loss reported in the sleep diaries, we did not find any associations with brain activity changes at a corrected level. However, a negative association with the rACC was visible at a lenient uncorrected threshold, which might not be too surprising given the moderate correlation between MCTQ and sleep diary. However, other state measures derived from the sleep diary could have proven more predictive. Two were tested in an exploratory fashion: intraindividual variability in sleep duration across the 7 days before scanning did not show an association with task activity in the mPFC. Sleep duration in the night before scanning, however, did demonstrate a moderate association, which suggests that the subthreshold findings in the sleep diary analysis may be driven by acute sleep loss in the night before scanning.

Importantly, in this study sleep deprivation was not imposed on participants artificially (Chuah et al., 2010; Yoo et al., 2007), thereby increasing the ecological validity of our results considerably. As such, our results have substantial implications: As most people are typically constrained to specific working hours and, as a consequence, sleep less than preferred, a vulnerability for maladaptive emotional processing may arise, also increasing risk for developing affective disorders (Gruber & Cassoff, 2014). One striking example is shift work. Two reviews concluded that sleep displacement leads to a broad variety of health risks, including metabolic, cardiovascular, and mental health disorders, although underlying mechanisms are still not well understood (James, Honn, Gaddameedhi, & Van Dongen, 2017; Kecklund & Axelsson, 2016).

There are several limitations to our study. First, the task was embedded in a longer experimental procedure, being preceded by, among other scans, a social-evaluative stress task. Oei et al. (2012) reported that acute stress has effects on brain activity during the same working memory task with emotional distraction as was used here. Because we did not include a nonstressed control group, we cannot rule out that responses to the stress induction contributed to our findings. However, based on the results of Oei et al. (2012), we assume that sleep loss effects, if influenced at all by stress, might have been amplified, as ventral affective areas were reported to be enhanced compared to a nonstress group. However, in contrast to Oei et al. (2012), we found task activity in the rACC, a region that was not reported to be influenced by stress. Also, there was no association between the area under the curve (with respect to increase) and task activity in the rACC, while subjective stress ratings significantly decreased after stress. It is thus unlikely that our results are merely stress-related. It is conceivable though that the prior induction of psychosocial stress weakened the associations with sleep loss through increasing alertness and arousal in our participants. However, we found no evidence that sleep loss was related to altered stress perception and thereby differential effects on the emotional working memory task.

Second, in the current study sleep loss was acquired with subjective measures (i.e., self-report questionnaire and sleep diary), which could have introduced a bias. In future studies, concomitant acquisition of objective sleep measures, such as output from actigraphic devices, should add to a clearer estimation of actual sleep disturbances (Jungquist, Pender, Klingman, & Mund, 2015).

Third, we scanned on Wednesday and Thursday evenings, which meant that there were only 3 or 4 work days before scanning. If we had scanned on Fridays instead of the two days before, results might have been positive for the sleep diary analysis as well, because participants would have had a higher chance of having accumulated sleep loss when including all 5 work days. Additionally, we have to consider that due to the scanning times between 5 and 10 p.m., sleep loss effects might have been influenced by the so-called wake maintenance zone (WMZ), which describes a certain time during evening hours before dim light melatonin onset, during which cognitive performance and alertness are increased instead of diminished by a sleep pressure accumulated during the day (Lavie, 1986). However, because our participants belonged to the group of later chronotypes and reported an average sleep onset time during working days at 0:27 a.m. (±57 min) and during free days even at 1:44 a.m. (±61 min), we assume that the WMZ might not have been reached by our participants. Furthermore, the WMZ seems to play a different role across cognitive domains. Whereas sustained attention and response inhibition were reported to improve during WMZ, performance in higher executive functioning, which would match our working memory task, was not affected (Zeeuw et al., 2018). Future studies might include the assessment of the dim light melatonin onset as an additional variable to be able to disentangle influences by the WMZ more in depth.

Fourth, because the stress task was a major component of our study and because it has been shown that females respond differently to stressors in terms of their cortisol responses (Liu et al., 2017), we chose to include males only. Therefore, our results on sleep loss only generalize to the male population, specifically to male late types.

The present findings revealed that naturally occurring sleep loss is associated with decreased activity in rostral ACC and dorsomedial PFC in a working memory task with emotional distracters, suggesting a modified interaction between brain regions that are associated with emotional and cognitive processing. Our results might reflect a mechanism by which accumulation of sleep loss over a prolonged period of time could enhance maladaptive emotional processing, thereby increasing vulnerability to develop affective disorders.
